# Diisopropyl­ammonium nitrite

**DOI:** 10.1107/S1600536812008574

**Published:** 2012-03-07

**Authors:** Ying-Chun Wang, Xu Jie

**Affiliations:** a College of Chemistry and Chemical Engineering, Southeast University, Nanjing 210096, People’s Republic of China

## Abstract

In the title mol­ecular salt, C_6_H_16_N^+^·NO_2_^−^, the cation forms two N—H⋯O hydrogen bonds to nearby nitrite anions which link the ionic units into chains propagating along the *b*-axis direction.

## Related literature

For a related structure, see: Xu (2012 ▶). For background to mol­ecular ferroelectric compounds, see: Fu *et al.* (2011 ▶).
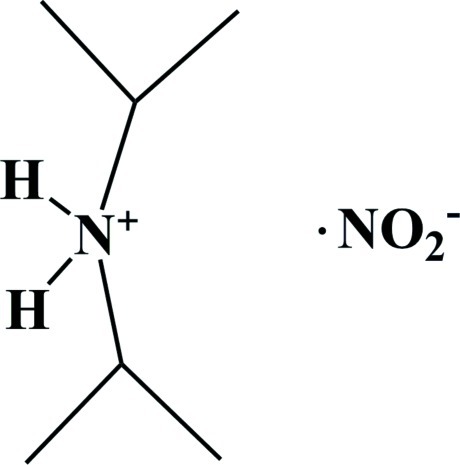


## Experimental

### 

#### Crystal data

C_6_H_16_N^+^·NO_2_^−^
*M* *_r_* = 148.21
Monoclinic, 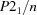

*a* = 8.2314 (16) Å
*b* = 7.7466 (15) Å
*c* = 14.583 (3) Å
β = 94.16 (3)°
*V* = 927.5 (3) Å^3^
*Z* = 4
Mo *K*α radiation
μ = 0.08 mm^−1^
*T* = 298 K
0.10 × 0.03 × 0.03 mm

#### Data collection

Rigaku Mercury2 CCD diffractometer
Absorption correction: multi-scan (*CrystalClear*; Rigaku, 2005 ▶) *T*_min_ = 0.910, *T*_max_ = 1.000
9189 measured reflections
2127 independent reflections
1130 reflections with *I* > 2σ(*I*)
*R*_int_ = 0.075

#### Refinement

*R*[*F* ^2^ > 2σ(*F* ^2^)] = 0.084
*wR*(*F* ^2^) = 0.285
*S* = 1.07
2127 reflections
95 parameters
15 restraints
H-atom parameters constrained
Δρ_max_ = 0.50 e Å^−3^
Δρ_min_ = −0.32 e Å^−3^

### 

Data collection: *CrystalClear* (Rigaku, 2005 ▶); cell refinement: *CrystalClear*; data reduction: *CrystalClear*; program(s) used to solve structure: *SHELXS97* (Sheldrick, 2008 ▶); program(s) used to refine structure: *SHELXL97* (Sheldrick, 2008 ▶); molecular graphics: *SHELXTL* (Sheldrick, 2008 ▶); software used to prepare material for publication: *SHELXTL*.

## Supplementary Material

Crystal structure: contains datablock(s) I, global. DOI: 10.1107/S1600536812008574/hb6657sup1.cif

Structure factors: contains datablock(s) I. DOI: 10.1107/S1600536812008574/hb6657Isup2.hkl

Supplementary material file. DOI: 10.1107/S1600536812008574/hb6657Isup3.cml

Additional supplementary materials:  crystallographic information; 3D view; checkCIF report

## Figures and Tables

**Table 1:** Hydrogen-bond geometry (Å, °)

*D*—H⋯*A*	*D*—H	H⋯*A*	*D*⋯*A*	*D*—H⋯*A*
N1—H1*E*⋯O1	0.90	1.90	2.800 (3)	174
N1—H1*D*⋯O1^i^	0.90	2.00	2.869 (3)	161

Symmetry code: (i) 
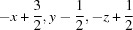
.

**Fig. 1.**
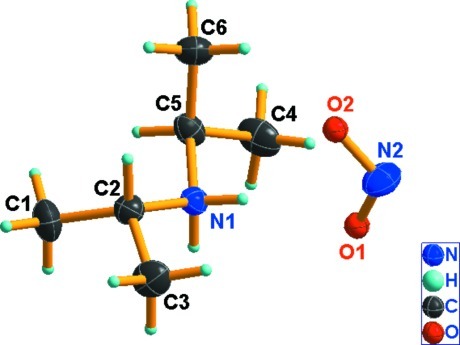
A view of the title compound with displacement ellipsoids drawn at the 30% probability level.

**Fig. 2.**
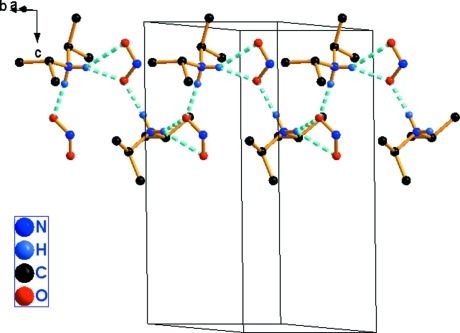
The crystal packing of the title compound showing the one-dimensionnal hydrogen bondings chain along the b axis (dashed line). H atoms not involved in hydrogen bonding (dashed line) have been omitted for clarity.

## supplementary crystallographic information

### Comment

Simple organic salts containig amino cations have attracted an attention as materials which display ferroelectric-paraelectric phase transitions (Fu *et al.*, 2011). As part of our ongonig studies in this area, (Xu, 2012), various amines have been studied and a series of new materials with this organic molecules have been elaborated. Herein we present the crystal structure of the title compound, di-isopropylammonium nitrite.

The asymmetric unit of the title compound contains one di-isopropylammonium cation and one NO_2_^-^ anion (Fig. 1). The amino N atom was protonated. The O-N-O bond angle of NO_2_- anion is 116.4 (4)°. And the other geometric parameters of the title compound are in the normal range.

In the crystal structure, all the ammonium H atoms are involved in intermolecular N—H···O H-bonding interactions with both the O atoms of the NO_2_^-^ anion (with N···O distances of 2.800 (3)Å and 2.869 (3)Å, respectively). These hydrogen bonds link the ionic units into a one-dimentional chain along the *b*-axis (Table 1 and Fig.2).

### Experimental

A mixture of di-isopropylamine (0.8 mmol), HCl (0.8 mmol) and NaNO_2_ (0.8 mmol) were dissolved in EtOH/distilled water (1:1 *v*/*v*) solvent. The solution was slowly evaporated in air affording colourless block-shaped crystals of the title compound.

### Refinement

All H atoms attached to C atoms were fixed geometrically and treated as riding with C-H = 0.98 Å(C methine) and C-H = 0.96 Å(C methyl) with *U*_iso_(H) = 1.2*U*_eq_(C methine) and *U*_iso_(H) = 1.5*U*_eq_(C methyl). The positional parameters of the H atoms (N) were refined freely. And in the last stage of the refinement, they were constrained with N—H = 0.90Å, and *U_iso_*(H)=1.2*U_eq_*(N).

### Crystal data

**Table:**

C_6_H_16_N^+^·NO_2_^−^	*F*(000) = 328
*M**_r_* = 148.21	*D*_x_ = 1.061 Mg m^−^^3^
Monoclinic, *P*2_1_/*n*	Mo *K*α radiation, λ = 0.71073 Å
Hall symbol: -P 2yn	Cell parameters from 2127 reflections
*a* = 8.2314 (16) Å	θ = 3.6–27.5°
*b* = 7.7466 (15) Å	µ = 0.08 mm^−^^1^
*c* = 14.583 (3) Å	*T* = 298 K
β = 94.16 (3)°	Block, colourless
*V* = 927.5 (3) Å^3^	0.10 × 0.03 × 0.03 mm
*Z* = 4	

### Data collection

**Table:**

Rigaku Mercury2 CCD diffractometer	2127 independent reflections
Radiation source: fine-focus sealed tube	1130 reflections with *I* > 2σ(*I*)
Graphite monochromator	*R*_int_ = 0.075
Detector resolution: 13.6612 pixels mm^-1^	θ_max_ = 27.5°, θ_min_ = 3.6°
CCD profile fitting scans	*h* = −10→10
Absorption correction: multi-scan (*CrystalClear*; Rigaku, 2005)	*k* = −10→10
*T*_min_ = 0.910, *T*_max_ = 1.000	*l* = −18→18
9189 measured reflections	

### Refinement

**Table:**

Refinement on *F*^2^	Primary atom site location: structure-invariant direct methods
Least-squares matrix: full	Secondary atom site location: difference Fourier map
*R*[*F*^2^ > 2σ(*F*^2^)] = 0.084	Hydrogen site location: inferred from neighbouring sites
*wR*(*F*^2^) = 0.285	H-atom parameters constrained
*S* = 1.07	*w* = 1/[σ^2^(*F*_o_^2^) + (0.1379*P*)^2^ + 0.0979*P*] where *P* = (*F*_o_^2^ + 2*F*_c_^2^)/3
2127 reflections	(Δ/σ)_max_ < 0.001
95 parameters	Δρ_max_ = 0.50 e Å^−^^3^
15 restraints	Δρ_min_ = −0.32 e Å^−^^3^

### Special details

**Table:**

Geometry. All esds (except the esd in the dihedral angle between two l.s. planes) are estimated using the full covariance matrix. The cell esds are taken into account individually in the estimation of esds in distances, angles and torsion angles; correlations between esds in cell parameters are only used when they are defined by crystal symmetry. An approximate (isotropic) treatment of cell esds is used for estimating esds involving l.s. planes.
Refinement. Refinement of F^2^ against ALL reflections. The weighted R-factor wR and goodness of fit S are based on F^2^, conventional R-factors R are based on F, with F set to zero for negative F^2^. The threshold expression of F^2^ > 2sigma(F^2^) is used only for calculating R-factors(gt) etc. and is not relevant to the choice of reflections for refinement. R-factors based on F^2^ are statistically about twice as large as those based on F, and R- factors based on ALL data will be even larger.

### Fractional atomic coordinates and isotropic or equivalent isotropic displacement parameters (Å^2^)

**Table:**

	*x*	*y*	*z*	*U*_iso_*/*U*_eq_	
N1	0.6713 (3)	0.1958 (3)	0.35567 (15)	0.0543 (7)	
H1D	0.6950	0.0825	0.3543	0.065*	
H1E	0.6890	0.2393	0.3000	0.065*	
C2	0.4930 (4)	0.2132 (4)	0.3690 (2)	0.0624 (9)	
H2A	0.4733	0.1702	0.4304	0.075*	
O1	0.7263 (3)	0.3533 (3)	0.18791 (17)	0.0931 (9)	
C5	0.7889 (4)	0.2792 (4)	0.4247 (2)	0.0686 (9)	
H5A	0.7676	0.4037	0.4255	0.082*	
N2	0.7438 (4)	0.2425 (4)	0.1320 (2)	0.0925 (9)	
O2	0.8050 (4)	0.2847 (5)	0.0648 (2)	0.1177 (10)	
C6	0.7724 (5)	0.2066 (6)	0.5196 (2)	0.0918 (12)	
H6A	0.6642	0.2277	0.5376	0.138*	
H6B	0.8500	0.2614	0.5626	0.138*	
H6C	0.7925	0.0846	0.5192	0.138*	
C3	0.3999 (4)	0.1012 (6)	0.2986 (3)	0.0909 (12)	
H3A	0.4409	−0.0148	0.3032	0.136*	
H3B	0.4133	0.1453	0.2381	0.136*	
H3C	0.2865	0.1017	0.3099	0.136*	
C4	0.9579 (5)	0.2488 (7)	0.3938 (3)	0.1037 (14)	
H4A	0.9700	0.3099	0.3374	0.156*	
H4B	0.9736	0.1275	0.3841	0.156*	
H4C	1.0374	0.2895	0.4402	0.156*	
C1	0.4425 (5)	0.3998 (5)	0.3631 (3)	0.0941 (13)	
H1A	0.5025	0.4643	0.4104	0.141*	
H1B	0.3280	0.4089	0.3710	0.141*	
H1C	0.4646	0.4452	0.3040	0.141*	

### Atomic displacement parameters (Å^2^)

**Table:**

	*U*^11^	*U*^22^	*U*^33^	*U*^12^	*U*^13^	*U*^23^
N1	0.0532 (15)	0.0552 (13)	0.0547 (13)	0.0026 (10)	0.0053 (10)	0.0051 (10)
C2	0.0558 (19)	0.0711 (19)	0.0611 (17)	0.0005 (14)	0.0101 (14)	0.0032 (14)
O1	0.1178 (19)	0.0879 (17)	0.0762 (14)	0.0013 (13)	0.0258 (13)	0.0032 (11)
C5	0.063 (2)	0.0582 (17)	0.082 (2)	−0.0024 (14)	−0.0121 (16)	0.0038 (15)
N2	0.116 (2)	0.0955 (18)	0.0689 (15)	0.0076 (14)	0.0293 (14)	−0.0001 (13)
O2	0.145 (2)	0.126 (2)	0.0854 (16)	0.0130 (16)	0.0308 (15)	0.0006 (14)
C6	0.102 (3)	0.110 (3)	0.061 (2)	0.003 (2)	−0.0109 (18)	−0.005 (2)
C3	0.061 (2)	0.114 (3)	0.097 (3)	−0.0143 (19)	0.0022 (19)	−0.014 (2)
C4	0.056 (2)	0.142 (4)	0.111 (3)	−0.011 (2)	−0.003 (2)	0.025 (3)
C1	0.068 (2)	0.089 (3)	0.125 (3)	0.0240 (19)	0.006 (2)	0.003 (2)

### Geometric parameters (Å, º)

**Table:**

N1—C5	1.492 (4)	C6—H6A	0.9600
N1—C2	1.500 (4)	C6—H6B	0.9600
N1—H1D	0.9000	C6—H6C	0.9600
N1—H1E	0.9000	C3—H3A	0.9600
C2—C1	1.505 (5)	C3—H3B	0.9600
C2—C3	1.509 (5)	C3—H3C	0.9600
C2—H2A	0.9800	C4—H4A	0.9600
O1—N2	1.200 (4)	C4—H4B	0.9600
C5—C6	1.510 (5)	C4—H4C	0.9600
C5—C4	1.512 (5)	C1—H1A	0.9600
C5—H5A	0.9800	C1—H1B	0.9600
N2—O2	1.181 (4)	C1—H1C	0.9600
			
C5—N1—C2	117.8 (2)	C5—C6—H6C	109.5
C5—N1—H1D	107.9	H6A—C6—H6C	109.5
C2—N1—H1D	107.9	H6B—C6—H6C	109.5
C5—N1—H1E	107.9	C2—C3—H3A	109.5
C2—N1—H1E	107.9	C2—C3—H3B	109.5
H1D—N1—H1E	107.2	H3A—C3—H3B	109.5
N1—C2—C1	110.4 (3)	C2—C3—H3C	109.5
N1—C2—C3	108.2 (2)	H3A—C3—H3C	109.5
C1—C2—C3	112.9 (3)	H3B—C3—H3C	109.5
N1—C2—H2A	108.4	C5—C4—H4A	109.5
C1—C2—H2A	108.4	C5—C4—H4B	109.5
C3—C2—H2A	108.4	H4A—C4—H4B	109.5
N1—C5—C6	111.1 (3)	C5—C4—H4C	109.5
N1—C5—C4	107.3 (3)	H4A—C4—H4C	109.5
C6—C5—C4	111.2 (3)	H4B—C4—H4C	109.5
N1—C5—H5A	109.1	C2—C1—H1A	109.5
C6—C5—H5A	109.1	C2—C1—H1B	109.5
C4—C5—H5A	109.1	H1A—C1—H1B	109.5
O2—N2—O1	116.4 (4)	C2—C1—H1C	109.5
C5—C6—H6A	109.5	H1A—C1—H1C	109.5
C5—C6—H6B	109.5	H1B—C1—H1C	109.5
H6A—C6—H6B	109.5		
			
C5—N1—C2—C1	63.0 (3)	C2—N1—C5—C6	60.4 (3)
C5—N1—C2—C3	−173.0 (3)	C2—N1—C5—C4	−177.8 (3)

### Hydrogen-bond geometry (Å, º)

**Table:**

*D*—H···*A*	*D*—H	H···*A*	*D*···*A*	*D*—H···*A*
N1—H1*E*···O1	0.90	1.90	2.800 (3)	174
N1—H1*D*···O1^i^	0.90	2.00	2.869 (3)	161

Symmetry code: (i) −*x*+3/2, *y*−1/2, −*z*+1/2.
